# Cannabis, Tobacco Use, and COVID-19 Outcomes

**DOI:** 10.1001/jamanetworkopen.2024.17977

**Published:** 2024-06-21

**Authors:** Nicholas B. Griffith, Timothy B. Baker, Brendan T. Heiden, Nina Smock, Giang Pham, Jingling Chen, Justin Yu, James Reddy, Albert M. Lai, Eric Hogue, Laura J. Bierut, Li-Shiun Chen

**Affiliations:** 1 Washington University School of Medicine, St Louis, Missouri; 2 Center for Tobacco Research and Intervention, School of Medicine and Public Health, University of Wisconsin, Madison; 3 Division of Cardiothoracic Surgery, Department of Surgery, Washington University School of Medicine, St Louis, Missouri; 4 Division of Public Health Sciences, Department of Surgery, Washington University School of Medicine, St Louis, Missouri; 5 Department of Psychiatry, Washington University School of Medicine, St Louis, Missouri; 6 Alvin J. Siteman Cancer Center at Barnes-Jewish Hospital, Washington University School of Medicine, St Louis, Missouri

## Abstract

**Importance:**

It is unclear whether cannabis use is associated with adverse health outcomes in patients with COVID-19 when accounting for known risk factors, including tobacco use.

**Objective:**

To examine whether cannabis and tobacco use are associated with adverse health outcomes from COVID-19 in the context of other known risk factors.

**Design, Setting, and Participants:**

This retrospective cohort study used electronic health record data from February 1, 2020, to January 31, 2022. This study included patients who were identified as having COVID-19 during at least 1 medical visit at a large academic medical center in the Midwest US.

**Exposures:**

Current cannabis use and tobacco smoking, as documented in the medical encounter.

**Main Outcomes and Measures:**

Health outcomes of hospitalization, intensive care unit (ICU) admission, and all-cause mortality following COVID-19 infection. The association between substance use (cannabis and tobacco) and these COVID-19 outcomes was assessed using multivariable modeling.

**Results:**

A total of 72 501 patients with COVID-19 were included (mean [SD] age, 48.9 [19.3] years; 43 315 [59.7%] female; 9710 [13.4%] had current smoking; 17 654 [24.4%] had former smoking; and 7060 [9.7%] had current use of cannabis). Current tobacco smoking was significantly associated with increased risk of hospitalization (odds ratio [OR], 1.72; 95% CI, 1.62-1.82; *P* < .001), ICU admission (OR, 1.22; 95% CI, 1.10-1.34; *P* < .001), and all-cause mortality (OR, 1.37, 95% CI, 1.20-1.57; *P* < .001) after adjusting for other factors. Cannabis use was significantly associated with increased risk of hospitalization (OR, 1.80; 95% CI, 1.68-1.93; *P* < .001) and ICU admission (OR, 1.27; 95% CI, 1.14-1.41; *P* < .001) but not with all-cause mortality (OR, 0.97; 95% CI, 0.82-1.14, *P* = .69) after adjusting for tobacco smoking, vaccination, comorbidity, diagnosis date, and demographic factors.

**Conclusions and Relevance:**

The findings of this cohort study suggest that cannabis use may be an independent risk factor for COVID-19–related complications, even after considering cigarette smoking, vaccination status, comorbidities, and other risk factors.

## Key Points

**Question:**

Is cannabis use associated with COVID-19 outcomes?

**Findings:**

In this cohort study of 72 501 patients diagnosed with COVID-19 in a large medical center, individuals who used cannabis had a higher risk of hospitalization and intensive care unit admission compared with those not using cannabis after controlling for other risk factors.

**Meaning:**

These findings suggest the need to evaluate the potential impact of cannabis use on COVID-19 outcomes given the growing legalized use of cannabis.

## Teaser

This cohort study evaluates whether cannabis use is associated with COVID-19–related outcomes, including hospitalization, intensive care unit admission, and mortality.

## Introduction

COVID-19 continues to be a public health concern, leading to morbidity and mortality. While nearly 76% of US adults have received at least 1 dose of the COVID-19 vaccine,^1^ many factors, including vaccine hesitancy and the emergence of new, more virulent strains of the SARS-CoV-2 virus, highlight the continued importance of identifying factors that contribute to poor outcomes from this viral illness. Several patient factors, including age, sex, race and ethnicity, and comorbidity burden,^2,3^ have been linked to more severe SARS-CoV-2 infection, but studies assessing the associations between modifiable risk factors—particularly substance use—and COVID-19 disease outcomes are more limited.^4,5,6,7,8,9^

Existing research on substance use and COVID-19 has been focused on cigarette smoking. Cigarette smoking has been found to be associated with more severe COVID-19 infection, including higher rates of disease progression,^9^ hospitalization, intensive care unit (ICU) admission, oxygen requirements during hospitalization, and mortality following COVID-19.^4,8^ There is early evidence suggesting the association of substance use disorder, alcohol use, and COVID-19 outcomes.^10,11,12^ In a large study using electronic health record (EHR) data, a substance use disorder diagnosis was associated with increased risk of COVID-19 and adverse outcomes, such as mortality and hospitalization.^11^ Another study suggests that there is an increased risk of COVID-19 breakthrough infections for people with substance use disorders.^11^ In a study of college students, high-risk alcohol use was associated with greater SARS-CoV-2 infection incidence, but not with COVID-19 outcomes.^10^

Despite the increasing availability of cannabis, research on cannabis and COVID-19 outcomes has been limited. With regards to cannabis use specifically, some evidence has suggested that people who use cannabis are more likely to contract COVID-19 and less likely to survive the virus than nonusers.^13^ Other evidence suggests a protective effect of cannabis use on COVID mortality.^14^ Given existing limited and conflicting findings, more evidence is needed on the association between substance use—particularly cannabis—and health outcomes following COVID-19 infection.

Clinical data available in EHRs can be a powerful tool for examining gaps in knowledge about the association of substance use with COVID-19 outcomes. The objective of this study is to examine a large sample of patients with COVID-19 to evaluate whether substance use (ie, tobacco smoking and cannabis use) is associated with several COVID-19–related outcomes, including hospitalization, ICU admission, and all-cause mortality. We hypothesized that tobacco smoking and cannabis use would be associated with worse outcomes following a COVID-19 infection.

## Methods

### Study Design

This retrospective cohort study extracted EHR data (Epic Systems) from all patients diagnosed with COVID-19 in a large academic medical center in the Midwest region of United States. Data collection was performed as part of the National Cancer Institute Cancer Center Cessation Initiative (C3I)^15^ and was approved by the Washington University Human Research Protection Office and Institutional Review Board. This project was granted a Health Insurance Portability and Accountability Act waiver and informed consent was waived due to use of de-identified data. Reporting of this study follows the Strengthening the Reporting of Observational Studies in Epidemiology (STROBE) reporting guideline.^16^

### Data Extraction

As part of the C3I, data extraction from our EHR platform was guided by the C3I COVID-19 Consortium led by UW Health (Madison).^17^ As part of this multi-institutional collaborative, EHR records for patients with COVID-19 encounters were queried in our EHR using a standardized extraction code created by the consortium.^18^

### Cohort Definition

This study included patients who were identified as having COVID-19 during at least 1 medical visit at the health care system between February 1, 2020, and January 31, 2022. COVID-19 cases were defined as meeting 1 of the following criteria: (1) an *International Classification of Diseases, Tenth Revision, Clinical Modification *(*ICD-10-CM*) diagnosis of COVID-19 (U07.1 or J12.82), (2) a positive result on a COVID-19 polymerase chain reaction test, (3) a positive result on a COVID-19 antibody test, or (4) a positive result on a COVID-19 antigen test. eFigure 1 in Supplement 1 shows the sample size filters of age range and missing data for inclusion in this study.

### Outcome Variables

The primary outcomes measured were hospitalization, ICU admission, and all-cause mortality. Posthospital mortality and other mortality outside the period a patient was hospitalized were included. Overall survival was also assessed using time-to-event analyses for those patients who had a documented date of mortality within our study period.

### Covariates

Demographic- and treatment-related covariates, including patient age, sex, race and ethnicity, health insurance coverage, and date of COVID-19 diagnosis were extracted from the EHR. Patient race and ethnicity were self-reported and documented by the rooming staff during all routine clinical encounters. The racial categories included American Indian or Alaska Native, Asian, Black, Other Pacific Islander, White, and other. Ethnic categories include Hispanic and non-Hispanic. Racial and ethnic categories other than Black and White were combined into the other category due to limited sample sizes. Race and ethnicity data were collected because they were included in routine clinical workflow and the EHR data extraction, and it was important to evaluate whether race and ethnicity were associated with COVID-19 outcomes along with other factors. Insurance status was coded yes if patients had Medicare, Medicaid, commercial insurance, or listed other as a form of insurance; it was coded no if patients were uninsured or self-pay.

Tobacco smoking and cannabis use were assessed using encounter-level data from the EHR. For smoking, patients self-reported current, former, or never smoking. Only patients with documented smoking status (current, former, or never) were included in the analyses (13% of patients were excluded due to missing smoking status). Current cannabis use was coded positive when positive marijuana use status was documented yes in the encounter medical record. Alcohol abuse in past 3 years and current vape use (e-cigarette or electronic nicotine delivery systems) were coded positive when such use was documented in the medical record. Vaping was considered an independent covariate and not part of tobacco or cannabis use. Discrete fields in the medical record, rather than free-text scanning using normal language processing, were extracted using the standardized extraction code created by the consortium.^18^

We also assessed various comorbidities (*ICD-10-CM* codes extracted from the discrete diagnoses documented within 3 years prior to COVID-19 infection) that have previously been shown to affect COVID-19 outcomes (Centers for Disease Control [CDC] Higher Risk Category),^19^ including history of malignant neoplasm, chronic kidney disease, chronic obstructive pulmonary disease, diabetes (type 1 or type 2), cardiovascular disease, obesity, and pregnancy. Obesity was defined as a body mass index (BMI; calculated as weight in kilograms divided by height in meters squared) greater than 30 for persons aged 20 years and older.^20^ Given that BMI is an unreliable measure of obesity in adolescents, the growth percentile classification scheme was used for individuals younger than 20 years, with those at the 95th percentile or greater classified as having obesity.

### Statistical Analysis

Cohort descriptive data were presented as numbers (proportions) with appropriate χ^2^ test statistics for categorical variables. To evaluate the association of substance use and COVID-19 outcomes (hospitalization, ICU admission, and mortality), we used both univariate and multivariable logistic regression models, adjusting for age, sex, race and ethnicity, health insurance status, comorbidities (composite), date of COVID-19 diagnosis, smoking status, and cannabis use. We also performed time-to-event analysis for overall survival using Cox proportional hazards regression models to evaluate the association of substance use and all-cause mortality. The proportional hazards assumption was checked using the scaled Shoenfeld residuals. Separate multivariable models were also constructed to assess comorbidities individually in sensitivity analyses. The association between alcohol abuse and vape use and risk of hospitalization was also assessed in a sensitivity analysis given the underdocumentation of these substance use risk factors. Given the hypotheses on the association of tobacco smoking and cannabis use on 3 major COVID-19 outcomes involving approximately 9 tests of association, we adjusted the significance thresholds from .05 to .005 for a Bonferroni correction to correct for multiple comparisons. All analyses were conducted using R version 3.5.3 (R Project for Statistical Computing), SAS version 9.4 (SAS Institute), and SPSS version 27 (IBM Corp).

## Results

### Sample Overview

This sample included 72 501 COVID-19 cases documented between February 1, 2020, and January 31, 2022. Study sample characteristics are shown in Table 1. Of those cases, 51 006 (70.4%) involved hospitalization, 4725 (6.5%) required an ICU visit, and 2717 (3.7%) resulted in mortality (Table 1). Patient age ranged from 12 years to older than 90 years, with a mean (SD) age of 48.9 (19.3) years. Overall, 43 315 patients (59.7%) identified as female and 29 186 (40.3%) as male; 20 003 (27.6%) were Black, and 50 438 (69.6%) were White. Most patients (49 881 [68.8%]) had at least 1 comorbidity that was identified as potentially affecting COVID-19 outcomes, the most common of which were obesity (35 029 [48.3%]), diabetes (13 457 [18.6%]), and cardiovascular disease (12 900 [17.8%]) (eTable 1 in Supplement 1). A total of 9710 patients (13.4%) reported current smoking, while 17 645 (24.4%) reported former smoking; 7060 patients (9.7%) reported current cannabis use.

**Table 1.** Distribution of Patient Characteristics and Outcomes Involving Hospitalization, ICU Admission, and Mortality

Characteristic	All patients with COVID-19, No. (column %)	Patients, No. (row %)		
		Hospitalization	ICU admission	Mortality
Total patients	72 501 (100)	51 006 (70.4)	4725 (6.5)	2717 (3.7)
Demographic characteristics				
Age, y				
12-35	20 971 (28.9)	13 240 (63.1)	423 (2.0)	58 (0.3)
36-50	16 704 (23)	11 072 (66.3)	595 (3.6)	179 (1.1)
51-65	18 666 (25.7)	13 571 (72.7)	1463 (7.8)	632 (3.4)
≥66	16 160 (22.3)	13 123 (81.2)	2244 (13.9)	1848 (11.4)
Sex				
Female	43 315 (59.7)	30 411 (70.2)	2097 (4.8)	1234 (2.8)
Male	29 186 (40.3)	20 595 (70.6)	2628 (9.0)	1483 (5.1)
Race				
White	50 438 (69.6)	32 725 (64.9)	3018 (5.9)	1850 (3.7)
Black	20 003 (27.6)	16 980 (84.9)	1519 (7.6)	770 (3.8)
Other^a^	2060 (2.8)	1301 (63.2)	188 (9.1)	97 (4.7)
Insurance				
Yes	68 748 (94.8)	47 953 (69.8)	4561 (6.6)	2632 (3.8)
No	3743 (5.2)	3053 (81.3)	164 (4.4)	85 (2.3)
Clinical factors				
Diagnosis date				
Jun 2020 and earlier	2319 (3.2)	2093 (90.3)	411 (17.7)	276 (11.9)
Jul-Dec 2020	19 750 (27.2)	15 942 (80.7)	1629 (8.3)	1041 (5.3)
Jan-Jun 2021	1025 (1.4)	8430 (82.2)	786 (7.7)	424 (4.1)
Jul 2021-Feb 2022	40 180 (55.4)	24 541 (61.1)	1899 (4.7)	976 (2.4)
COVID-19 vaccine before diagnosis				
No	53 091 (73.2)	41 336 (77.9)	4079 (7.7)	2340 (4.4)
Yes	19 410 (26.8)	9670 (49.8)	646 (3.3)	377 (1.9)
Comorbidity^b^				
No	22 620 (31.2)	13 720 (60.7)	557 (2.5)	185 (0.8)
Yes	49 881 (68.8)	37 286 (74.7)	4168 (8.4)	2532 (5.1)
Substance use				
Smoking status				
Never	45 137 (62.3)	29 969 (66.4)	2314 (5.1)	1172 (2.6)
Former	17 654 (24.4)	13 323 (75.5)	1782 (10.1)	1238 (7.0)
Current	9710 (13.4)	7714 (79.4)	629 (6.5)	307 (3.2)
Cannabis^c^				
No	65 441 (90.3)	45 257 (69.2)	4247 (6.5)	2535 (3.9)
Yes	7060 (9.7)	5749 (81.4)	478 (6.8)	182 (2.6)

Abbreviation: ICU, intensive care unit.

^a^ The other category includes American Indian or Alaska Native, Asian, Other Pacific Islander, and all other racial backgrounds documented in the electronic health record.

^b^ Comorbidities in US Centers for Disease Control and Prevention tier 1.

^c^ Cannabis use was defined as any cannabis use ever documented in any encounters at the time of COVID-19 diagnosis.

### Association of Tobacco Smoking With COVID-19 Outcomes

#### Hospitalization

**Figure 1.**
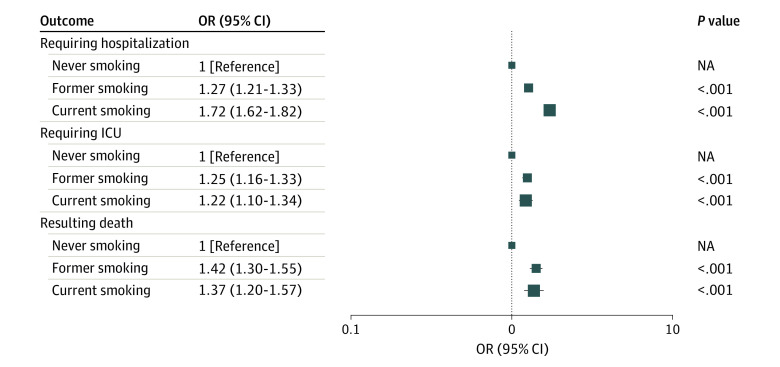
Tobacco Smoking and COVID-19 Outcomes of Hospitalization, Intensive Care Unit (ICU) Admission, and Mortality Box sizes indicate patient groups from smallest (never smoking) to largest (current smoking). NA indicates not applicable; OR, odds ratio.

A total of 51 006 patients (70.4%) were hospitalized (Table 1). Compared with never smoking, both current (odds ratio [OR], 1.72; 95% CI, 1.62-1.82; *P* < .001) and former (OR, 1.27; 95% CI, 1.21-1.33; *P* < .001) smoking were associated with an increased risk of hospitalization following COVID-19, adjusted for age, sex, race and ethnicity, insurance status, any comorbidity, cannabis use, diagnosis date, and first dose of COVID-19 vaccination before diagnosis (Table 2 and Figure 1). Ever smoking (vs never smoking) was associated with greater risk of hospitalization in multivariable logistic regression, adjusted for age, sex, race and ethnicity, insurance status, any comorbidity, cannabis use, diagnosis date, and first dose of COVID-19 vaccination before diagnosis (OR, 1.41; 95% CI, 1.36-1.47; *P* < .001).

**Table 2.** Associations of Patient Characteristics With Outcomes of Hospitalization, ICU Admission, and Mortality Among 72 501 Individuals with COVID-19^a^

Characteristic	Hospitalization		ICU admission		Mortality	
	OR (95% CI)	*P* value	OR (95% CI)	*P* value	OR (95% CI)	*P* value
Demographic						
Age, y						
12-35	1 [Reference]	NA	1 [Reference]	NA	1 [Reference]	NA
36-50	1.17 (1.11-1.22)	<.001	1.59 (1.40-1.81)	<.001	3.29 (2.46-4.47)	<.001
51-65	1.69 (1.61-1.77)	<.001	3.39 (3.03-3.81)	<.001	9.68 (7.43-12.86)	<.001
≥66	3.18 (3.00-3.37)	<.001	6.38 (5.69-7.17)	<.001	34.9 (26.9-46.2)	<.001
Sex						
Female	1 [Reference]	NA	1 [Reference]	NA	1 [Reference]	NA
Male	0.86 (0.83-0.90)	<.001	1.69 (1.59-1.80)	<.001	1.43 (1.32-1.55)	<.001
Race						
White	1 [Reference]	NA	1 [Reference]	NA	1 [Reference]	NA
Black	3.12 (2.98-3.27)	<.001	1.45 (1.35-1.55)	<.001	1.30 (1.19-1.43)	<.001
Other^b^	1.11 (1.01-1.23)	.04	2.23 (1.89-2.63)	<.001	2.05 (1.63-2.56)	<.001
Insurance						
Yes	1 [Reference]	NA	1 [Reference]	NA	1 [Reference]	NA
No	1.71 (1.56-1.87)	<.001	0.94 (0.79-1.10)	.45	1.63 (1.28-2.05)	<.001
Clinical						
Diagnosis date						
Jun 2020 and earlier	1 [Reference]	NA	1 [Reference]	NA	1 [Reference]	NA
Jul-Dec 2020	0.66 (0.57-0.76)	<.001	0.48 (0.42-0.54)	<.001	0.44 (0.38-0.52)	<.001
Jan-Jun 2021	0.77 (0.66-0.90)	<.001	0.47 (0.41-0.54)	<.001	0.38 (0.32-0.45)	<.001
Jul 2021-Feb 2022	0.35 (0.31-0.41)	<.001	0.43 (0.38-0.49)	<.001	0.36 (0.30-0.42)	<.001
COVID-19 vaccine before diagnosis						
No	1 [Reference]	NA	1 [Reference]	NA	1 [Reference]	NA
Yes	0.36 (0.35-0.38)	<.001	0.40 (0.37-0.45)	<.001	0.42 (0.36-0.47)	<.001
Any comorbidity^c^						
No	1 [Reference]	NA	1 [Reference]	NA	1 [Reference]	NA
Yes	1.59 (1.53-1.65)	<.001	2.39 (2.17-2.62)	<.001	3.28 (2.82-3.84)	<.001
Smoking status						
Never	1 [Reference]	NA	1 [Reference]	NA	1 [Reference]	NA
Former	1.27 (1.21-1.33)	<.001	1.25 (1.16-1.33)	<.001	1.42 (1.30-1.55)	<.001
Current	1.72 (1.62-1.82)	<.001	1.22 (1.10-1.34)	<.001	1.37 (1.20-1.57)	<.001
Cannabis^d^						
No	1 [Reference]	NA	1 [Reference]	NA	1 [Reference]	NA
Yes	1.80 (1.68-1.93)	<.001	1.27 (1.14-1.41)	<.001	0.97 (0.82-1.14)	.69

Abbreviations: ICU, intensive care unit; NA, not applicable; OR, odds ratio.

^a^ Logistic regression models included covariates age, sex, race and ethnicity, insurance status, any comorbidity, smoking status, cannabis use, diagnosis date, and first dose of COVID-19 vaccination before diagnosis.

^b^ The other category includes American Indian or Alaska Native, Asian, Other Pacific Islander, and all other racial backgrounds documented in the electronic health record.

^c^ Comorbidities in the US Centers for Disease Control and Prevention tier 1.

^d^ Cannabis use was defined as any marijuana use ever documented in any encounter at the time of COVID-19 diagnosis.

#### ICU Admission

A total of 4725 patients (6.5%) were admitted to the ICU (Table 1). Both current smoking (OR, 1.22; 95% CI, 1.10-1.34; *P* < .001) and former smoking (OR, 1.25; 95% CI, 1.16-1.33; *P* < .001), compared with never smoking, were associated with an increased risk of ICU admission following COVID-19, adjusted for covariates (Table 2 and Figure 1). Ever smoking (vs never smoking) was associated with greater odds of ICU admission adjusted for age, sex, race and ethnicity, insurance status, any comorbidity, cannabis use, diagnosis date, and first dose of COVID-19 vaccination before diagnosis (OR, 1.24; 95% CI, 1.16-1.32; *P* < .001).

#### Mortality

A total of 2717 patients (3.7%) died (Table 1). Current smoking (OR, 1.37; 95% CI, 1.20-1.57; *P* < .001) and former smoking (OR, 1.42; 95% CI, 1.30-1.55; *P* < .001) were both associated with increased risk of all-cause mortality following COVID-19, adjusted for covariates (Table 2 and Figure 1). Ever smoking (vs never smoking) was associated with more mortality in multivariable logistic regression adjusted for age, sex, race and ethnicity, insurance status, any comorbidity, cannabis use, diagnosis date, and first dose of COVID-19 vaccination before diagnosis (OR, 1.41; 95% CI, 1.30-1.53; *P* < .001). We reached similar results on the association of smoking and the hazard of mortality using Cox regression (eTable 2 in Supplement 1).

#### Mortality by Age

**Figure 2.**
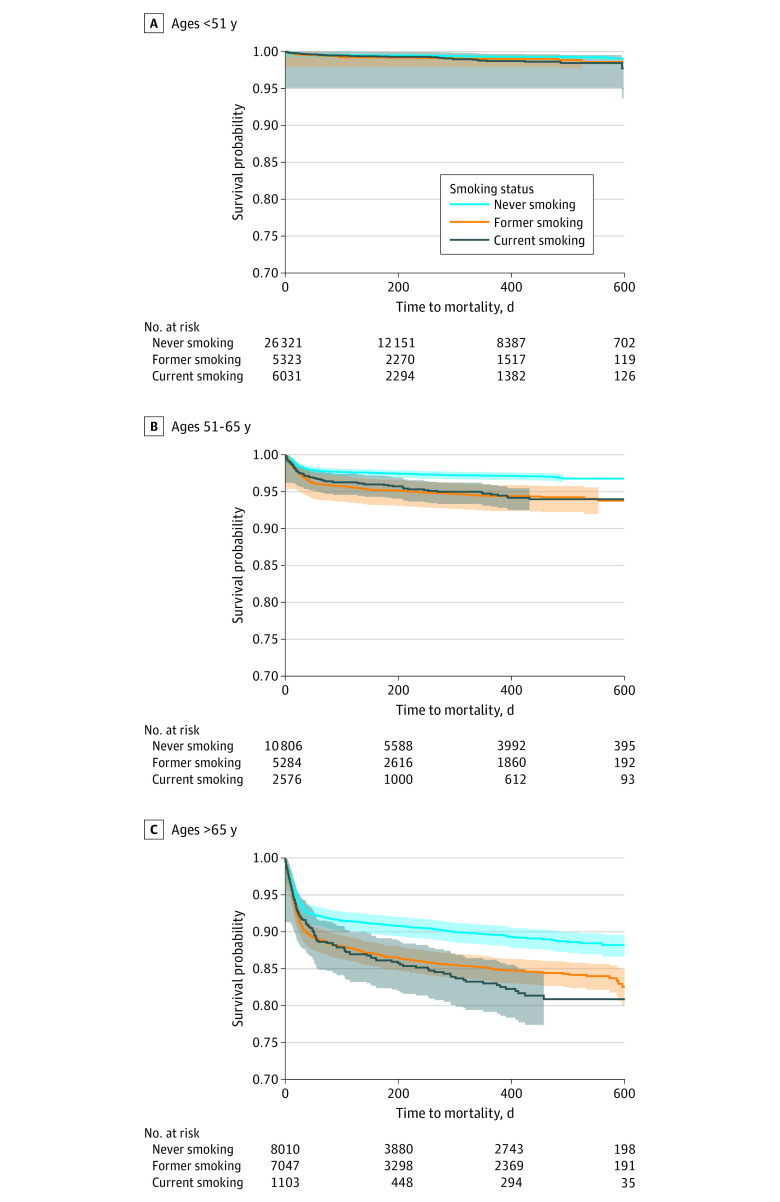
Association of Tobacco Smoking and COVID-19–Related Hazard of Mortality, Stratified by Age Shaded areas indicate 95% CIs.

For the oldest age group (>65 years), patients with former (hazard ratio [HR], 1.46; 95% CI, 1.32-1.60; *P* < .001) or current (HR, 1.57; 95% CI, 1.31-1.86; *P* < .001) smoking had a faster progression to all-cause mortality than those with never smoking (Figure 2C; eTable 3 in Supplement 1). We reached similar results in Cox regression models adjusted for covariates (eFigure 2 in Supplement 1).

#### Comparing Current and Former Smoking

In addition to the previous comparisons with never smoking, we compared current vs former smoking. We found a significantly higher probability of hospitalization (OR, 1.28; 95% CI, 1.20-1.38; *P* < .001) but not ICU admission (OR, 0.98; 95% CI, 0.88-1.10; *P* = .78) or all-cause mortality (OR, 0.99; 95% CI, 0.86-1.14; *P* = .87) among current compared with former smokers (eTable 4 in Supplement 1).

### Association of Cannabis Use With COVID-19 Outcomes

#### Hospitalization

**Figure 3.**
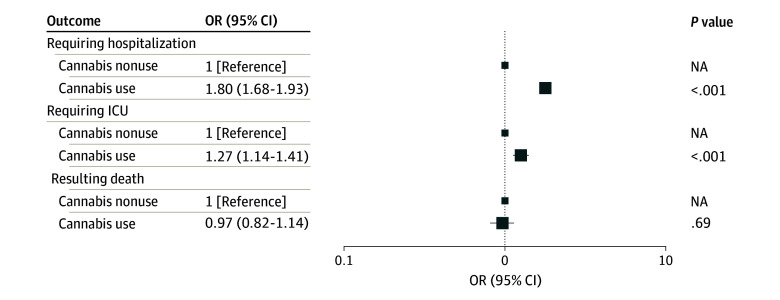
Cannabis Use and COVID-19 Outcomes Box sizes indicate patient groups, with smallest indicating cannabis nonuse and largest indicating cannabis use. NA indicates not applicable; OR, odds ratio.

Cannabis current use was documented in 7060 patients (9.7%) (Table 1). Cannabis use was significantly associated with an increased risk of hospitalization following COVID-19, adjusted for covariates including tobacco use (OR, 1.80; 95% CI, 1.68-1.93; *P* < .001) (Table 2 and Figure 3).

#### ICU Admission

Similarly, cannabis use was associated with an increased risk of ICU admission following COVID-19. After adjusting for covariates, the OR was 1.27 (95% CI, 1.14-1.41; *P* < .001) (Table 2 and Figure 3).

#### Mortality

However, cannabis use was not associated with an increase all-cause mortality following COVID-19. The OR was 0.97 (95% CI, 0.82-1.14; *P* = .69) (Table 2 and Figure 3C).

### Adjusting for Comorbidities

eTable 1 in Supplement 1 shows the frequency of comorbid conditions in this sample. Further analysis was done to see whether any of the 7 comorbidities defined by the CDC as a tier 1 comorbidity were individually associated with COVID-19 outcomes. These analyses found that these comorbid conditions increased the risk of COVID-19 outcomes. We reached similar results regarding tobacco and cannabis use when adjusting for each of the comorbid conditions (eTable 5 in Supplement 1). We have conducted additional analyses with refined categories for covariates (insurance types, ethnicity, age groups) and reached similar results (data not shown).

### Tobacco, Cannabis Use, and Receipt of COVID-19 Vaccine

The characteristics of patients who received vaccination before diagnosis are shown in eTable 6 in Supplement 1. A total of 19 410 patients (26.8%) received a COVID-19 vaccine before the diagnosis. Since vaccination before diagnosis is associated with COVID-19 outcomes, we evaluated factors associated with vaccination before diagnosis. Current and former smoking was associated with a lower probability of receiving a COVID-19 vaccine before diagnosis (current smoking: OR, 0.60; 95% CI, 0.56-0.63; *P* < .001; former smoking: OR, 0.93; 95% CI, 0.89-0.97; *P* < .001), while adjusting for other variables (eTable 7 in Supplement 1). Cannabis use was not significantly associated with receipt of the COVID-19 vaccine (OR, 1.04; 95% CI, 0.98-1.11; *P* = .21) when adjusting other variables including smoking status.

### Other Substance Use and COVID-19 Outcomes

EHR data are limited for other substance use. Alcohol abuse in the past 3 years was documented in 250 patients (0.3%), and current vape use was documented in 1384 patients (1.9%) (eTable 1 in Supplement 1). These variables are known to be severely underrecorded in the EHR, therefore limiting statistical power significantly.^17^ Despite these limits, the OR for alcohol abuse and hospitalization was greater than 1, but the *P* value was greater than our prespecified level of statistical significance (OR, 3.34; 95% CI, 2.08-5.69; *P* = .01) (eTable 8 in Supplement 1). Likewise, the OR for vaping and hospitalization was greater than 1, but the *P* value did not meet our prespecified level of statistical significance (OR, 1.20; 95% CI, 1.06-1.37; *P* = .006). There was not sufficient data on alcohol and vaping to evaluate their association with COVID-19–related ICU admission and mortality (eTable 8 in Supplement 1).

## Discussion

Given the continued risk of COVID-19, this study extends current evidence on the potential impact of substance use on COVID-19 outcomes. Using EHR data from a large medical center, we provide further evidence on the association of tobacco use with an increased risk of hospitalization, ICU admission, and all-cause mortality related to COVID-19 infection. Importantly, we present new evidence suggesting that cannabis use may be associated with an increased risk of hospitalization and ICU admission following COVID-19, while adjusting for other factors, such as tobacco smoking, comorbidities, and COVID-19 vaccination before diagnosis.

Our findings may help clarify the complex multidimensional impact of tobacco smoking on COVID-19 outcomes. While some research indicates a protective association between smoking and COVID-19 severity (referred to as a smoker’s paradox^21^), most research demonstrates that tobacco smoking is associated with an increased risk of symptomatic infection with SARS-CoV-2 as well as an increased risk of disease progression.^4,6,7^ Interestingly, some studies indicate increased severity of COVID-19 infection in individuals who formerly smoked, including higher rates of hospitalization, ICU admission, oxygen requirement during hospitalization, and in-hospital mortality, but not in those who currently smoke.^4^ Here, we presented evidence using EHR data of more than 72 000 COVID-19 cases and showed that current and former smoking status were both associated with poor COVID-19 outcomes, characterized by an increased risk of hospitalization, ICU admission, and all-cause mortality following COVID-19, compared with those who have never smoked, after considering other risk factors.

Given the rising availability of cannabis, these findings also contribute to the existing limited research on potential effects of cannabis use on COVID-19 outcomes.^7,22^ A recent study shows that any substance use disorder was associated with worse COVID-19 outcomes; however, the design did not have large enough sample sizes to evaluate the association of specific substances, such as cannabis, with COVID-19 severity.^12^ Another study suggested a protective association of cannabis with COVID-19 mortality^23^; however, their sample size was smaller and unidentified collider bias could be an important source of paradoxical associations.^23^ In this study, we provide evidence of an association between cannabis use and poorer COVID-19 outcomes characterized by both an increased risk of hospitalization and ICU admission.

Furthermore, we presented preliminary data on the association between other forms of substance use, including alcohol abuse and vaping, and COVID-19 outcomes. There is currently very limited research examining the association of vaping (ie, e-cigarette use) and the severity of COVID-19 outcomes in patients.^24^ In our study, we presented preliminary findings that vaping may be associated with an increased risk of hospitalization, despite limited documentation regarding vape use in the EHR data. Similarly, there is research on increased alcohol consumption during the COVID-19 pandemic,^25^ while little is known about the association between alcohol use and COVID-19 outcomes. One study found that alcohol use was associated with an increased risk of SARS-CoV-2 infection in a small cohort of college students.^10^ We present a potential association between alcohol abuse and increased risk of hospitalization following COVID-19 infection. Notably, further studies are needed, as our findings were limited by small sample sizes and limited documentation within our EHR database.

### Limitations

This study has limitations. First, the study spanned 24 months (February 2020 to January 2022), which may have included significantly different SARS-CoV-2 disease manifestations due to the emergence of new variants, time-varying policies related to universal masking and lockdowns, and the introduction of the COVID-19 vaccine in December 2020. To reduce this concern, we included date of diagnosis and vaccination before diagnosis in our multivariable models to reduce the confounding effect of different outcomes related to time. However, caution should still be exercised when interpreting our results due to the potential for persistent confounding. Second, EHR data are limited by relying on patient self-report of substance use and subsequent documentation by medical staff. Therefore, substance use data quality within EHR often suffers from variable reporting and missing documentation. The best existing measure in the EHR data, current cannabis use, is a very crude measure without specific details on cannabis type, frequency, or recency. We have tried to reduce this bias by using data from all available hospital encounters. These findings should be viewed with caution because a detection bias is possible if heavier marijuana users were more likely to have that status documented and were at greater risk for morbidity. This study sample representing patients who required health care services for COVID-19 may not be representative of the general population regarding their substance use. Furthermore, there were insufficient data on the types of tobacco products (eg, cigars, pipe) because product types were often undocumented and nonmandatory in most medical encounters. We acknowledge that health care system–wide EHR-based data suffer from these biases despite the large set of clinical data they represent. Third, additional factors, such as mental health status, were not included in this investigation given their potential impacts on substance use and health outcomes. Additionally, these findings are based on information in our EHR data. Although our EHR tracks patient mortality outcomes beyond our health care system, it is possible that the data do not fully capture outcomes for patients who sought care or died at another institution.

## Conclusions

This cohort study found that both current and former smoking were associated with an increased risk among patients with COVID-19 for hospitalization, ICU admission, and all-cause mortality. These associations remained after adjusting for demographic and comorbidity factors. Specifically, older patients who reported current or former smoking showed a faster progression to all-cause mortality than those who reported never smoking. In addition, cannabis use was associated with an increased risk of hospitalization and ICU admission among patients with COVID-19. Our preliminary data also suggest a need for further investigation into whether other forms of substance use, including nicotine and cannabis vaping and alcohol abuse, are associated with worse COVID-19 outcomes. Overall, this research calls for further investigation into the associations of tobacco and cannabis use with COVID-19 outcomes. Given the recent legalization of recreational marijuana use in more states, including the area served by this academic medical center, further research may aid in guiding interventions, such as substance use prevention and treatment, that would benefit patient outcomes moving forward in the COVID-19 pandemic and the associated heath consequences it will have in our communities.

## Notes

Supplement 1. **eTable 1.** Comorbidity Details and Other Clinical Characteristics of Patients with COVID-19 and Outcomes Involving Hospitalization, ICU Admission, and Mortality **eTable 2.** Associations of Patient Characteristics and the Hazard of Mortality **eTable 3.** Association of Tobacco Smoking and COVID-19–Related Hazard of Mortality, Stratified by Age **eTable 4.** Specific Comparison Between Patients With Current Smoking vs Patients With Former Smoking **eTable 5.** Associations of Patient Characteristics (Including Comorbidity Details) and Outcomes of Hospitalization, ICU Admission, and Mortality **eTable 6.** Characteristics of Patients With COVID-19 and Outcomes of Receiving COVID-19 Vaccine Before Diagnosis **eTable 7.** Associations of Patient Characteristics and the Outcome of Receiving the COVID-19 Vaccine Before Diagnosis Using Logistic Regression **eTable 8.** Associations of Patient Characteristics (Including Vaping and Alcohol Use) With Outcomes of Hospitalization, ICU Admissions, and Mortality **eFigure 1.** Flowchart Demonstrating the Data Filter and Selection Process **eFigure 2**. Association of Tobacco Smoking and COVID-19–Related Hazard of Mortality, Stratified by Age, Adjusted for Other Patient Characteristics

Supplement 2.
Data Sharing Statement
