# Error in Figure

**DOI:** 10.1001/jamanetworkopen.2024.27937

**Published:** 2024-07-15

In the Original Investigation titled, “Cannabis, Tobacco Use, and COVID-19 Outcomes,”^1^ published June 21, 2024, there was an error in Figure 3. The *P* value for the odds ratio for cannabis use and resulting death should have read .69. This article has been corrected.^1^

## References

[1] Griffith NB, Baker TB, Heiden BT, . Cannabis, tobacco use, and COVID-19 outcomes. JAMA Netw Open. 2024;7(6):e2417977. doi:10.1001/jamanetworkopen.2024.17977 38904961 PMC11193123

