# Supplementary material for: Cannabis, Tobacco Use, and COVID-19 Outcomes
Source: JAMA Netw Open. 2024 Jun 21;7(6):e2417977. doi: 10.1001/jamanetworkopen.2024.17977 (PMC11193123; doi:10.1001/jamanetworkopen.2024.17977)
Supplement: Supplement 1. — eTable 1. Comorbidity Details and Other Clinical Characteristics of Patients with COVID-19 and Outcomes Involving Hospitalization, ICU Admission, and Mortality eTable 2. Associations of Patient Characteristics and the Hazard of Mortality eTable 3. Association of Tobacco Smoking and COVID-19–Related Hazard of Mortality, Stratified by Age eTable 4. Specific Comparison Between Patients With Current Smoking vs Patients With Former Smoking eTable 5. Associations of Patient Characteristics (Including Comorbidity Details) and Outcomes of Hospitalization, ICU Admission, and Mortality eTable 6. Characteristics of Patients With COVID-19 and Outcomes of Receiving COVID-19 Vaccine Before Diagnosis eTable 7. Associations of Patient Characteristics and the Outcome of Receiving the COVID-19 Vaccine Before Diagnosis Using Logistic Regression eTable 8. Associations of Patient Characteristics (Including Vaping and Alcohol Use) With Outcomes of Hospitalization, ICU Admissions, and Mortality eFigure 1. Flowchart Demonstrating the Data Filter and Selection Process eFigure 2. Association of Tobacco Smoking and COVID-19–Related Hazard of Mortality, Stratified by Age, Adjusted for Other Patient Characteristics [file jamanetwopen-e2417977-s001.pdf]

Supplemental Online Content

Griffith N, Baker TB, Heiden BT, et al. Cannabis, tobacco use, and COVID-19 outcomes. *JAMA Netw Open.* 2024;7(6):e2417977. doi:10.1001/jamanetworkopen.2024.17977

eTable 1. Comorbidity Details and Other Clinical Characteristics of Patients with COVID-19 and Outcomes Involving Hospitalization, ICU Admission, and Mortality

eTable 2. Associations of Patient Characteristics and the Hazard of Mortality

eTable 3. Association of Tobacco Smoking and COVID-19–Related Hazard of Mortality, Stratified by Age

eTable 4. Specific Comparison Between Patients With Current Smoking vs Patients With Former Smoking

eTable 5. Associations of Patient Characteristics (Including Comorbidity Details) and Outcomes of Hospitalization, ICU Admission, and Mortality

eTable 6. Characteristics of Patients With COVID-19 and Outcomes of Receiving COVID-19 Vaccine Before Diagnosis

eTable 7. Associations of Patient Characteristics and the Outcome of Receiving the COVID-19 Vaccine Before Diagnosis Using Logistic Regression

eTable 8. Associations of Patient Characteristics (Including Vaping and Alcohol Use) With Outcomes of Hospitalization, ICU Admissions, and Mortality

eFigure 1. Flowchart Demonstrating the Data Filter and Selection Process

eFigure 2 . Association of Tobacco Smoking and COVID-19–Related Hazard of Mortality, Stratified by Age, Adjusted for Other Patient Characteristics

This supplemental material has been provided by the authors to give readers additional information about their work.

© 2024 Griffith N et al. *JAMA Network Open.*

eTable 1. Comorbidity Details and Other Clinical Characteristics of Patients with COVID-19 and Outcomes Involving Hospitalization, ICU Admission, and Mortality

Characteristic	All COVID-19 cases (n)	Hospitalization (n, row %)	ICU (n, row %)	Mortality (n, row %)
	72501	51006 (70.4)	4725 (6.5)	2717 (3.7)
**Comorbidity***				
**Comorbidity (Any)** [a]				
No	22620	13720 (60.7)	557 (2.5)	185 (.8)
Yes	49881	37286 (74.7)	4168 (8.4)	2532 (5.1)
**Chronic Kidney Disease**				
No	63579	43217 (68.0)	2848 (4.5)	1367 (2.2)
Yes	8922	7789 (87.3)	1877 (21.0)	1350 (15.1)
**Any Chronic Lung Disease**				
No	66345	45749 (69.0)	3711 (5.6)	2001 (3.0)
Yes	6156	5257 (85.4)	1014 (16.5)	716 (11.6)
**Obesity**				
No	37472	25453 (67.9)	2311 (6.2)	1509 (4.0)
Yes	35029	25553 (72.9)	2414 (6.9)	1208 (3.4)
**Any Cancer**				
No	63814	44262 (69.4)	3757 (5.9)	1845 (2.9)
Yes	8687	6744 (77.6)	968 (11.1)	872 (10.0)
**Pregnancy**				
No	69004	48439 (70.2)	4672 (6.8)	2707 (3.9)
Yes	3497	2567 (73.4)	53 (1.5)	10 (.3)
**Any Cardiovascular Disease**				
No	59601	40183 (67.4)	2619 (4.4)	1173 (2.0)
Yes	12900	10823 (83.9)	2106 (16.3)	1544 (12.0)
**Diabetes Mellitus**				
No	59044	40016 (67.8)	2772 (4.7)	1514 (2.6)
Yes	13457	10990 (81.7)	1953 (14.5)	1203 (8.9)
**Substance use**				
**Smoking Status**				
Never	45137	29969 (66.4)	2314 (5.1)	1172 (2.6)
Former	17654	13323 (75.5)	1782 (10.1)	1238 (7.0)
Current	9710	7714 (79.4)	629 (6.5)	307 (3.2)
**Cannabis** [b]				
No	65441	45257 (69.2)	4247 (6.5)	2535 (3.9)
Yes	7060	5749 (81.4)	478 (6.8)	182 (2.6)
**Vaping** [c]				
No	71117	50006 (70.3)	4648 (6.5)	2692 (3.8)
Yes	1384	1000 (72.3)	77 (5.6)	25 (1.8)
**Alcohol abuse** [d]				
No	72251	50774 (70.3)	4673 (6.5)	2697 (3.7)
Yes	250	232 (92.8)	52 (20.8)	20 (8.0)

Note: [a] Comorbidities in CDC Tier 1

© 2024 Griffith N et al. *JAMA Network Open.*

[b] Cannabis use was defined as any marijuana ever use documented on any encounters at the time of COVID-19 diagnosis.
[c] Vaping was defined as any e-cigarette use documented on any encounters at the time of COVID-19 diagnosis.
[d] Alcohol abuse was defined as any alcohol abuse documented on any encounters at the time of COVID-19 diagnosis.

© 2024 Griffith N et al. *JAMA Network Open.*

eTable 2. Associations of Patient Characteristics and the Hazard of Mortality (n=72,501)

Characteristic	Overall mortality	
	Hazard Ratio (95% CI)	*P*-value
**Demographic**		
**Age**		
12 to 35	reference	
36 to 50	3.29 (2.45-4.43)	<.0001
51 to 65	9.60 (7.31-12.60)	<.0001
66 and above	32.7 (25.0-42.8)	<.0001
**Sex**		
Female	reference	
Male	1.39 (1.29-1.50)	<.0001
**Race**		
White	reference	
African-American or Black	1.27 (1.17-1.39)	<.0001
Other	1.95 (1.59-2.39)	<.0001
**Insurance**		
Yes	reference	
No	1.63 (1.30-2.04)	<.0001
**Clinical predictor**		
**Diagnosis date**		
Before Jun 2020	reference	
Jul-Dec 2020	.52 (.45-.60)	<.0001
Jan-Jun 2021	.47 (.40-.55)	<.0001
Jul 2021-Feb 2022	.61 (.53-.71)	<.0001
**COVID-19 vaccine before diagnosis**		
No	reference	
Yes	.45 (.40-.51)	<.0001
**Comorbidity [a] (any)**		
No	reference	
Yes	3.16 (2.72-3.68)	<.0001
**Substance use**		
**Smoking Status**		
Never	reference	
Former	1.39 (1.28-1.50)	<.0001
Current	1.36 (1.20-1.55)	<.0001
**Cannabis [b]**		
No	reference	
Yes	.96 (.82-1.12)	.59

Note: Cox regression model included covariates such as age, sex, race, insurance status, any comorbidity, smoking status, Cannabis use, diagnosis date and first dose of COVID-19 vaccination before diagnosis.
[a] Comorbidities in CDC Tier 1
[b] Cannabis use was defined as any marijuana ever use documented on any encounters at the time of COVID-19 diagnosis.

© 2024 Griffith N et al. *JAMA Network Open.*

eTable 3. Association of Tobacco Smoking and COVID-19–Related Hazard of Mortality, Stratified by Age

(A) Patients with age < 51

	Survival (95% CI)		
	30 days	3 months	6 months
Never smoking	1.00 (1.00-1.00)	1.00 (.99-1.00)	.99 (.99-1.00)
Former smoking	1.00 (.99-1.00)	.99 (.99-1.00)	.99 (.99-.99)
Current smoking	1.00 (.99-1.00)	.99 (.99-1.00)	.99 (.99-.99)

(B) Patients with age 51-65

	Survival (95% CI)		
	30 days	3 months	6 months
Never smoking	.98 (.98-.99)	.98 (.98-.98)	.98 (.97-.98)
Former smoking	.97 (.97-.97)	.96 (.95-.96)	.95 (.95-.96)
Current smoking	.97 (.97-.98)	.96 (.95-.97)	.96 (.95-.96)

(C) Patients with age > 65

	Survival probability (95% CI)		
	30 days	3 months	6 months
Never smoking	.94 (.93-.94)	.92 (.91-.92)	.91 (.90-.91)
Former smoking	.91 (.90-.91)	.88 (.87-.89)	.87 (.86-.88)
Current smoking	.90 (.88-.92)	.87 (.85-.89)	.86 (.85-.89)

(A) Among patients with age <51: 26,321 never smoking, 5,323 former smoking status, 6,031 current smoking status. Former and current smoking (vs. never smoking) are not significantly associated with higher hazard of more mortality in Cox regression (HR, 1.41; 95% CI, 1.00-1.997; *P*=.0503; HR= 1.51, 95% CI, 1.088-2.088; *P*=.014). Shoenfeld p=0.0038.

(B) Among patients with age 51-65: 10,806 never smoking status, 5,284 former smoking status, 2,576 current smoking status. Former and current smoking (vs. never smoking) are associated with higher hazard of more mortality in Cox regression (HR, 1.94; 95% CI, 1.63-2.301; *P*<.0001; HR, 1.78; 95% CI, 1.42-2.22; *P*<.0001). Shoenfeld p=0.37.

(C) Among patients with age >65: 4,334 never smoking status, 3,725 former smoking status, 467 current smoking status. Former and current smoking (vs. never smoking) are associated with higher hazard of more mortality in Cox regression (HR, 1.46; 95% CI, 1.32-1.60; *P*<.0001; HR, 1.57; 95% CI, 1.31-1.86; *P*<.0001). Shoenfeld p=0.052.

© 2024 Griffith N et al. *JAMA Network Open.*

eTable 4. Specific Comparison Between Patients With Current Smoking vs Patients With Former Smoking

Characteristic	Hospitalization		ICU		Mortality	
	**OR (95% CI)**	***P*-value**	**OR (95% CI)**	***P*-value**	**OR (95% CI)**	***P*-value**
**Demographics**						
**Age**						
12 to 35	reference		reference		reference	
36 to 50	.84 (.76-.92)	.000158	1.54 (1.24-1.93)	.000103	3.15 (1.90-5.54)	< .0001
51 to 65	1.27 (1.16-1.40)	< .0001	3.49 (2.87-4.28)	<.0001	11.71 (7.38-19.94)	< .0001
66 and above	2.47 (2.22-2.74)	< .0001	6.13 (5.02-7.53)	< .0001	36.67 (23.14-62.37)	< .0001
**Sex**						
Female	reference		reference		reference	
Male	.94 (.88-1.00)	.047	1.60 (1.46-1.75)	<.0001	1.29 (1.15-1.44)	< .0001
**Race**						
White	reference		reference		reference	
African-American or Black	3.13 (2.88-3.41)	<.0001	1.53 (1.39-1.69)	< .0001	1.25 (1.10-1.41)	.00057
Other	1.17 (.95-1.46)	.14	2.29 (1.76-2.96)	< .0001	1.85 (1.31-2.57)	.00031
**Insurance**						
Yes	reference		reference		reference	
No	1.69 (1.46-1.97)	< .0001	.90 (.70-1.15)	.41	1.47 (1.03-2.04)	.029
**Clinical predictor**						
**Diagnosis date**						
Before Jun 2020	reference		reference		reference	
Jul-Dec 2020	.62 (.47-.81)	.00081	.55 (.46-.66)	< .0001	.47 (.38-.58)	<.0001
Jan-Jun 2021	.73 (.54-.97)	.033	.53 (.43-.64)	< .0001	.41 (.32-.51)	<.0001
Jul 2021-Feb 2022	.31 (.23-.41)	< .0001	.41 (.34-.50)	< .0001	.32 (.26-.40)	<.0001
**COVID-19 vaccine before diagnosis**						
No	reference		reference			
Yes	.36 (.34-.39)	< .0001	.51 (.45-.58)	< .0001	.53 (.45-.63)	< .0001
**Comorbidity [a] (any)**						
No	reference		reference		reference	
Yes	1.47 (1.37-1.58)	< .0001	2.52 (2.17-2.93)	< .0001	3.28 (2.82-3.84)	< .0001
**Substance use**						
**Smoking Status**						
Former	reference		reference		reference	
Current	1.28 (1.20-1.38)	< .0001	.98 (.88-1.10)	.78	.99 (.86-1.14)	.87
**Cannabis [b]**						
No	Reference		reference		reference	
Yes	1.81 (1.65-2.00)	< .0001	1.35 (1.19-1.54)	< .0001	1.04 (.86-1.25)	.65

[a] Comorbidities in CDC Tier 1
[b] Cannabis use was defined as any marijuana ever use documented on any encounters at the time of COVID-19 diagnosis.

© 2024 Griffith N et al. *JAMA Network Open.*

eTable 5. Associations of Patient Characteristics (Including Comorbidity Details) and Outcomes of Hospitalization, ICU Admission, and Mortality (n=72,501)

Characteristic	Hospitalization		ICU		Mortality	
	OR (95% CI)	*P*-value	OR (95% CI)	*P*-value	OR (95% CI)	*P*-value
**Demographic**						
**Age**						
12 to 35	reference		reference		reference	
36 to 50	1.21 (1.15-1.27)	< .0001	1.49 (1.31-1.70)	< .0001	3.23 (2.41-4.40)	< .0001
51 to 65	1.61 (1.53-1.70)	< .0001	2.59 (2.30-2.92)	< .0001	7.38 (5.63-9.85)	< .0001
66 and above	2.51 (2.35-2.67)	< .0001	3.66 (3.23-4.16)	< .0001	17.9 (13.7-23.9)	< .0001
**Sex**						
Female	reference		reference		reference	
Male	.86 (.82-.89)	< .0001	1.56 (1.46-1.66)	< .0001	1.29 (1.19-1.41)	< .0001
**Race**						
White	reference		reference		reference	
African-American or Black	3.01 (2.88-3.16)	< .0001	1.20 (1.11-1.28)	< .0001	1.09 (.98-1.20)	.099
Other	1.10 (1.00-1.22)	.062	2.22 (1.88-2.61)	< .0001	2.13 (1.69-2.67)	< .0001
**Insurance**						
Yes	reference		reference		reference	
No	1.80 (1.65-1.97)	< .0001	1.05 (.88-1.23)	.605	1.98 (1.56-2.50)	< .0001
**Clinical**						
**Diagnosis date**						
Before Jun 2020	reference		reference			
Jul-Dec 2020	.68 (.58-.78)	< .0001	.50 (.44-.57)	< .0001	.47 (.40-.55)	< .0001
Jan-Jun 2021	.79 (.68-.92)	.0025	.49 (.43-.57)	< .0001	.39 (.33-.47)	< .0001
Jul 2021-Feb 2022	.36 (.31-.42)	< .0001	.47 (.41-.54)	< .0001	.39 (.33-.46)	< .0001
**COVID-19 vaccine before diagnosis**						
No	reference		reference		reference	
**Comorbidity** [a]						
Chronic Kidney Disease	1.80 (1.67-1.94)	< .0001	2.55 (2.37- 2.75)	< .0001	2.82 (2.57-3.09)	< .0001
Any Chronic Lung Disease	1.54 (1.42-1.67)	< .0001	1.48 (1.35-1.61)	< .0001	1.41 (1.27-1.56)	< .0001
Obesity	1.15 (1.11-1.19)	< .0001	1.10 (1.03-1.17)	.0035	.89 (.82-.97)	.0086
Any Cancer	1.29 (1.21-1.37)	< .0001	1.07 (.99-1.17)	.095	1.62 (1.48-1.78)	< .0001
Pregnancy	1.83 (1.68-2.00)	< .0001	.75 (.56-.98)	.043	.61 (.30-1.10)	.13
Any Cardiovascular Disease	1.46 (1.37-1.55)	< .0001	1.51 (1.40- 1.63)	< .0001	1.75 (1.59-1.93)	< .0001
Diabetes Mellitus	1.30 (1.23-1.38)	< .0001	1.54 (1.44-1.66)	< .0001	1.31 (1.19-1.43)	< .0001
**Substance use**						
**Smoking Status**						
Never	reference		reference		reference	
Former	1.18 (1.13-1.24)	< .0001	1.08 (1.00-1.16)	.0505	1.18 (1.07-1.29)	.00052
Current	1.63 (1.54-1.73)	< .0001	1.05 (.95-1.17)	.33	1.12 (.97-1.29)	.12
**Cannabis** [b]						
No	reference		reference		reference	
Yes	1.78 (1.66-1.90)	< .0001	1.21 (1.09-1.35)	.00046	.90 (.76-1.05)	.19

© 2024 Griffith N et al. *JAMA Network Open.*

Note: Logistic regression models included covariates such as age, sex, race, insurance status, 7 comorbidities in CDC Tier 1, smoking status, Cannabis use, diagnosis date and first dose of COVID-19 vaccination before diagnosis.

[a] Comorbidities in CDC Tier 1

[b] Cannabis use was defined as any marijuana ever use documented on any encounters at the time of COVID-19 diagnosis.

© 2024 Griffith N et al. *JAMA Network Open.*

eTable 6. Characteristics of Patients With COVID-19 and Outcomes of Receiving COVID-19 Vaccine Before Diagnosis

	All COVID-19 cases (n)	Receiving vaccine before diagnosis (n, row %)
**Study Population**	72501	19410 (26.8)
**Demographics**		
**Age**		
12-35	20971	4529 (27.1)
36-50	16704	4553 (24.4)
51-65	18666	5204 (32.2)
66 and above	16160	5124 (31.7)
**Sex**		
Female	43315	12413 (28.7)
Male	29186	6997 (24.0)
**Race**		
White	50438	14731 (29.2)
African American	20003	4073 (20.4)
Other	2060	606 (29.4)
**Insurance**		
Yes	68748	19085 (27.8)
No	3743	325 (8.7)
**Clinical predictor**		
**Diagnosis date**		
Before Jun 2020	2319	0 (.0)
Jul-Dec 2020	19750	53 (.27)
Jan-Jun 2021	10252	1037 (10.1)
Jul 2021-Feb 2022	40180	18320 (45.6)
**Comorbidity** [a]		
No	22620	5740 (25.4)
Yes	49881	13670 (27.4)
**Substance use**		
**Smoking Status**		
Never	45137	1918 (27.4)
Former	17654	5108 (28.9)
Current	9710	1918 (19.8)
**Cannabis** [b]		
No	65441	17758 (27.1)
Yes	7060	1652 (23.4)

[a] Comorbidities in CDC Tier 1
[b] Cannabis use was defined as any marijuana ever use documented on any encounters at the time of COVID-19 diagnosis.

eTable 7. Associations of Patient Characteristics and the Outcome of Receiving the COVID-19 Vaccine Before Diagnosis Using Logistic Regression (n=72,501)

Characteristic	COVID-19 vaccine before diagnosis	
	**Odds Ratio (95% CI)**	***P*-value**
**Demographic**		
**Age**		
12 to 35	reference	
36 to 50	1.47 (1.39-1.55)	< .0001
51 to 65	1.59 (1.50-1.67)	< .0001
66 and above	1.88 (1.78-1.99)	< .0001
**Sex**		
Female	reference	
Male	.79 (.76-.82)	< .0001
**Race**		
White	reference	
African-American or Black	.67 (.64-.70)	< .0001
Other	1.08 (.97-1.20)	.17
**Insurance**		
Yes	reference	
No	.26 (.23-.29)	< .0001
**Clinical**		
**Diagnosis date** [a]		
2020-2021	reference	
2022	6.66 (6.41-6.93)	< .0001
**Comorbidity** [b]		
No	reference	
Yes	1.02 (.97-1.06)	.48
**Substance use**		
**Smoking**		
Never smoker	reference	
Former Smoker	.93 (.89-.97)	.0007
Current smoker	.60 (.56-.63)	< .0001
**Cannabis** [c]		
No	reference	
Yes	1.04 (.98-1.11)	.21

Note: COVID-19 vaccine was defined as receiving the first dose before COVID-19 diagnoses. Logistic regression model included covariates such as age, sex, race, insurance status, any comorbidity, smoking status, Cannabis use, diagnosis date.
[a] Diagnosis date was consolidated into 2 groups: 2020-2021 and 2022 due the lack of the vaccination status in this period. COVID-19 vaccine was available to the public in 2021.
[b] Comorbidities in CDC Tier 1
[c] Cannabis use was defined as any marijuana ever use documented on any encounters at the time of COVID-19 diagnosis.

© 2024 Griffith N et al. *JAMA Network Open.*

eTable 8. Associations of Patient Characteristics (Including Vaping and Alcohol Use) With Outcomes of Hospitalization, ICU Admissions, and Mortality (n=72,501)

**Characteristic**	**Hospitalization**	
	**Odds Ratio (95% CI)**	***P*-value**
**Demographic**		
**Age**		
12 to 35	reference	
36 to 50	1.17 (1.11-1.23)	< .0001
51 to 65	1.69 (1.61-1.78)	< .0001
66 and above	3.20 (3.02-3.39)	< .0001
**Sex**		
Female	reference	
Male	.86 (.83-.90)	< .0001
**Race**		
White	reference	
African-American or Black	3.13 (2.99-3.28)	< .0001
Other	1.11 (1.01-1.23)	.035
**Insurance**		
No	reference	
Yes	1.71 (1.57-1.88)	< .0001
**Clinical**		
**Diagnosis date**		
Before June 2020	reference	
Jul-Dec 2020	.66 (.57-.77)	< .0001
Jan-Jun 2021	.77 (.66-.90)	< .00083
Jul 2021-Feb 2022		
**COVID-19 vaccine before diagnosis**		
No	reference	
Yes	.36 (.35-.38)	<.0001
**Comorbidity [a]**		
No	reference	
Yes	1.59 (1.53-1.65)	< .0001
**Substance use**		
**Smoking Status**		
Never	reference	
Former	1.26 (1.21-1.32)	< .0001
Current	1.69 (1.59-1.79)	< .0001
**Cannabis [b]**		
No	reference	
Yes	1.78 (1.66-1.91)	< .0001
**Vaping**		
No	reference	
Yes	1.20 (1.06-1.37)	.0055
**Alcohol abuse**		
No	reference	
Yes	3.34 (2.08-5.69)	.011

© 2024 Griffith N et al. *JAMA Network Open.*

Note: Logistic regression model included covariates such as age, sex, race, insurance status, any comorbidity, smoking status, Cannabis use, diagnosis date and first dose of COVID-19 vaccination before diagnosis.
[a] Comorbidities in CDC Tier 1
[b] Cannabis use was defined as any marijuana ever use documented on any encounters at the time of COVID-19 diagnosis.

© 2024 Griffith N et al. *JAMA Network Open.*

eFigure 1. Flowchart Demonstrating the Data Filter and Selection Process

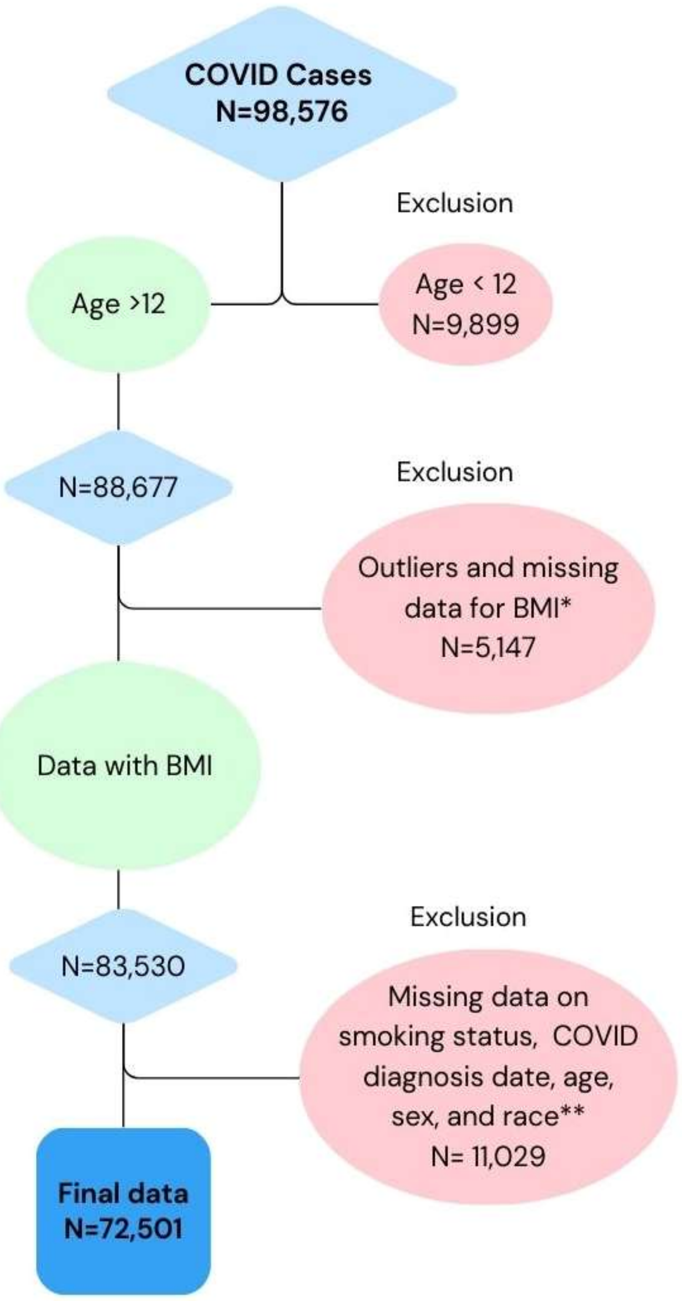

*Outliers for Body Mass Index (BMI) were assessed by filtering out weight < 10kg and height < 24 cm.
** Out of 83,530 cases, 7 had missing sex, 37 had missing race, 3,577 had missing age, 1 had missing COVID diagnosis date, and 6,721 had missing smoking status.

© 2024 Griffith N et al. *JAMA Network Open.*

eFigure 2: Association of Tobacco Smoking and COVID-19–Related Hazard of Mortality, Stratified by Age, Adjusted for Other Patient Characteristics

(A) Patients with age < 51

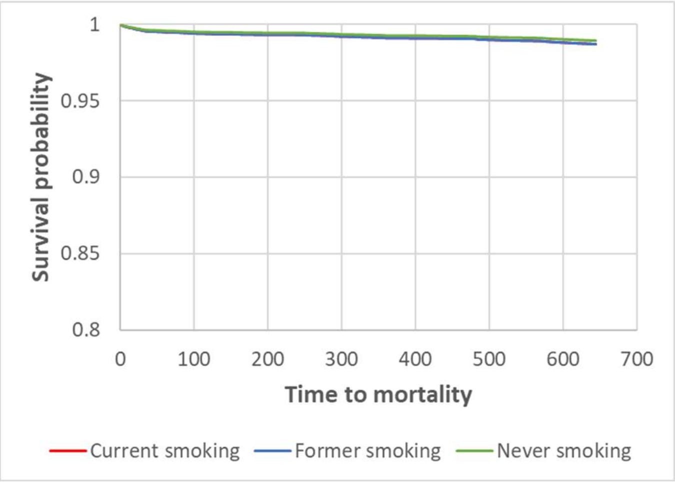

	Survival (95% CI)		
	30 days	3 months	6 months
Never smoking	1.00 (1.00-1.00)	1.00 (.99-1.00)	.99 (.99-1.00)
Former smoking	1.00 (.99-1.00)	.99 (.99-1.00)	.99 (.99-1.00)
Current smoking	1.00 (.99-1.00)	.99 (.99-1.00)	.99 (.99-1.00)

(B) Patients with age 51-65

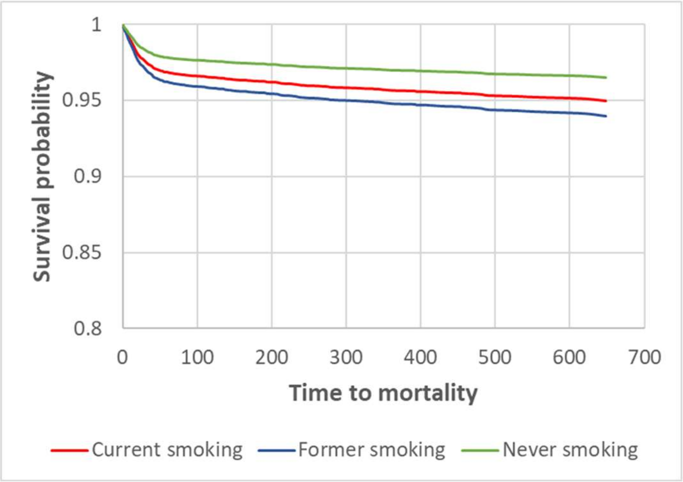

	Survival (95% CI)		
	30 days	3 months	6 months
Never smoking	.98 (.98-0.99)	.98 (.97-.98)	.97(.97-.98)
Former smoking	.97 (.97-0.97)	.96 (.95-.96)	.96 (.95-.96)
Current smoking	.98 (.97-0.98)	.97 (.96-.97)	.96 (.96-.97)

(C) Patients with age > 65

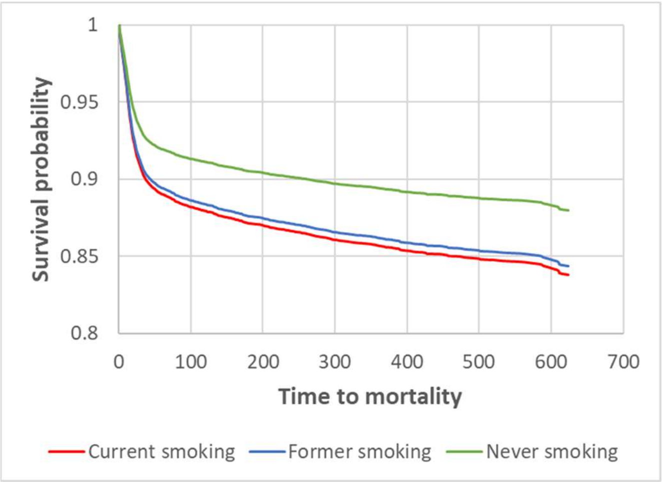

	Survival (95% CI)		
	30 days	3 months	6 months
Never smoking	.93 (.93-.94)	.91 (.91-.92)	.91 (.90-.91)
Former smoking	.91 (.90-.92)	.89 (.88-.89)	.88 (.87-.88)
Current smoking	.90 (.89-.92)	.88 (.87-.90)	.87 (.85-.89)

(A) Among patients with age <51: 26,321 never smoking status, 5,323 former smoking status, 6,031 current smoking status. Former and current smoking (vs. never smoking) are not associated with higher hazard of more mortality in Cox regression (HR, 1.22; 95% CI, .86-1.73; *P*=.27; HR, 1.22; 95% CI, .87-1.73; *P*=.25), adjusted for sex, race, insurance status, any comorbidity, smoking status, cannabis use, diagnosis date and first dose of COVID vaccination before diagnosis.

(B) Among patients with age 51-65: 10,806 never smoking status, 5,284 former smoking status, 2,576 current smoking status. Former and current smoking (vs. never smoking) are associated with higher hazard of more mortality in Cox regression (HR, 1.76; 95% CI, 1.48-2.10; *P*<.0001; HR, 1.46; 95% CI, 1.16-1.84; *P*=.0013), adjusted for sex, race, insurance status, any comorbidity, smoking status, cannabis use, diagnosis date and first dose of COVID vaccination before diagnosis.

(C) Among patients with age >65: 4,334 never smoking status, 3,725 former smoking status, 467 current smoking status. Former smoking (vs. never smoking) are associated with higher hazard of more mortality in Cox regression but current smoking are not (HR, 1.34; 95% CI, 1.21-1.47; *P*<.0001; HR, 1.39; 95% CI, 1.16-1.66; *P*=.0003), adjusted for sex, race, insurance status, any comorbidity, smoking status, cannabis use, diagnosis date and first dose of COVID vaccination before diagnosis.

© 2024 Griffith N et al. *JAMA Network Open.*
