# Supplementary material for: Cannabis, Tobacco Use, and COVID-19 Outcomes
Source: JAMA Netw Open. 2024 Jun 21;7(6):e2417977. doi: 10.1001/jamanetworkopen.2024.17977 (PMC11193123; doi:10.1001/jamanetworkopen.2024.17977)
Supplement: Supplement 2. — Data Sharing Statement [file jamanetwopen-e2417977-s002.pdf]

# Data Sharing Statement

Griffith. Cannabis, Tobacco Use, and COVID-19 Outcomes. *JAMA Netw Open*. Published June 21, 2024. doi:10.1001/jamanetworkopen.2024.17977

**Data**
**Data available:** Yes
**Data types:** Deidentified participant data
**How to access data:** De-identified data will be made available to anyone who submits an approved request to the coordinating center at the University of Wisconsin-Madison. Requests may be made through the following link. https://uwmadison.co1.qualtrics.com/jfe/form/SV_41sykjxDStxBOWa The principal investigator, Li-Shiun Chen, had full access to all the data in the study and takes responsibility for the integrity of the data and the accuracy of the data analysis.
**When available:** With publication

**Supporting Documents**
**Document types:** None

**Additional Information**
**Who can access the data:** De-identified data will be made available to anyone who submits an approved request to the coordinating center at the University of Wisconsin-Madison. Requests may be made through the following link. https://uwmadison.co1.qualtrics.com/jfe/form/SV_41sykjxDStxBOWa
**Types of analyses:** Data will be made available for any analysis approved by the coordinating center at University of Wisconsin Madison. From the data-request web-page: "Data requests will be reviewed by the C3I Coordinating Center Team and at least 2 members of the C3I Expert Panel, typically within two weeks of receipt of the completed proposal and data request form."
**Mechanisms of data availability:** From the data-request web-page: "Upon approval of the request, the Coordinating Center will provide a Data Use Agreement for completion by the primary proposer." & "If you have any questions about the C3I data, please reach out to Dr. Jennifer Bird, the Project Scientist, at jebird@wisc.edu." Any additional mechanisms may be made known by contacting the Coordinating Center The principal investigator, Li-Shiun Chen, had full access to all the data in the study and takes responsibility for the integrity of the data and the accuracy of the data analysis.
**Any additional restrictions:** From the data-request web-page: "Upon review of the request, the Coordinating Center will inform the primary proposer: If the manuscript/presentation proposal is approved, provisionally approved, or requires revisions If the data requested can be shared If necessary, what alternatives may be feasible Coordinating Center capacity to provide analytic support In what form the data can be transferred (e.g., aggregate, by site, software to be used, etc.)"
